# Positive airway pressure therapy for obstructive sleep apnea in patients with Osteogenesis imperfecta: a prospective pilot study

**DOI:** 10.1186/s12891-020-03932-9

**Published:** 2021-01-11

**Authors:** Heidi Arponen, Adel Bachour, Leif Bäck, Helena Valta, Antti Mäkitie, Outi Mäkitie, Janna Waltimo-Sirén

**Affiliations:** 1 Department of Oral and Maxillofacial Diseases, University of Helsinki, P.O. Box 41, FI-00014 Helsinki, Finland; 2 Research Program for Clinical and Molecular Metabolism, Faculty of Medicine, University of Helsinki, Helsinki, Finland; 3 Sleep Unit, Heart and Lung Center, Helsinki University Hospital and University of Helsinki, Helsinki, Finland; 4 Department of Otorhinolaryngology – Head and Neck Surgery, Helsinki University Hospital, University of Helsinki, Helsinki, Finland; 5 Children´s Hospital and Pediatric Research Center, University of Helsinki and Helsinki University Hospital, Helsinki, Finland; 6 Folkhälsan Institute of Genetics and University of Helsinki, Helsinki, Finland; 7 Center for Molecular Medicine, Karolinska Institutet, and Clinical Genetics, Karolinska University Hospital, Stockholm, Sweden; 8 Department of Oral and Maxillofacial Diseases, Helsinki University Hospital, Helsinki, Finland; 9 Institute of Dentistry, University of Turku, Turku, Finland; 10 City of Turku, Division of Welfare, Turku, Finland

**Keywords:** Osteogenesis imperfecta, Sleep apnea, Fatigue, Sleepiness, Depression, PAP therapy, Adherence

## Abstract

**Background:**

Obstructive sleep apnea (OSA) is prevalent in individuals with Osteogenesis imperfecta (OI). To date, no study has investigated treatment of OSA in adult individuals with OI using positive airway pressure (PAP). This observational pilot study examined the adherence of adults with OI to treatment of OSA with PAP therapy, and the evolution of self-experienced sleepiness and depression symptoms before and after treatment.

**Methods:**

We included 20 patients, with a mean age of 51 years, who represented varying severity of OI and displayed an apnea and hypopnea index ≥ 5 /sleeping hour as recorded by an overnight polysomnography. PAP therapy was proposed to all patients. Epworth Sleepiness Scale (ESS) questionnaire to evaluate daytime sleepiness, and a validated self-rating depression questionnaire to identify possible depression, were completed prior to PAP therapy and repeated after a minimum of one year. The datasets supporting the conclusions of this article are included within the article.

**Results:**

From the 20 patients, 15 initiated PAP therapy, and two patients later interrupted it. The mean PAP follow-up period was 1230 days. At baseline, an abnormally high ESS score was reported by 29% of the respondents, and an abnormally high number of symptoms suggesting depression by 29%. Follow-up questionnaires were completed by 60% of the patients, of whom 83% were adherent to PAP treatment. ESS score and depression symptoms did not decrease significantly with PAP therapy.

**Conclusions:**

Patients with OI accepted well PAP therapy and remained compliant. Sleepiness and depression persisted unaltered despite good PAP adherence. These unexpectedly poor improvements in symptoms by PAP therapy may be due to subjective depression symptoms and the complexity of factors underlying persisting sleepiness in OI. Further research is needed to confirm this novel finding.

## Background

Osteogenesis imperfecta (OI) is a group of rare genetic disorders, in which mutations impair normal synthesis, assembly, folding, posttranslational modification, secretion, or structure of type I collagen. The succedent detriment of collagen-rich tissues leads to typical clinical manifestations, such as bone fragility, hyperextensibility of ligaments, short stature, hearing impairment, and a tendency to progressive skeletal deformity of long bones, spine and cranial base [1, 2]. Phenotypic classification of OI into five types covers a severity range from mild non-deforming (type I) to severe (type III) and lethal (type II) [3].

In addition to a variety of somatic changes, patients with OI experience pain, persisting daytime sleepiness, and fatigue [3–5]. Our previous studies indicate that one of the factors contributing to fatigue in OI might be sleep apnea [6, 7]. Previous studies [4, 7] have reported in adults with OI a prevalence of sleep apnea exceeding that in normal population [8].

In general, individuals with obstructive sleep apnea (OSA) show a high prevalence of depression [9], and depression and tiredness are highly correlated traits [10]. Treatment of OSA with positive airway pressure (PAP) has been documented to result in subjective alleviation of daytime sleepiness, fatigue and depression [11].

The purpose of this study was firstly, to evaluate acceptance of and adherence to positive airway pressure (PAP) therapy among adult OI patients, and secondly, to evaluate the effect of PAP therapy on self-experienced daytime sleepiness and depression.

## Methods

### Study population

In 2015, we invited all 151 patient members, over the age of 16 years, of the Finnish OI Society, to participate in a study exploring the quality of sleep and its relation to daytime well-being [6, 7]. The study protocol was approved by the Research Ethic Board of Helsinki University Hospital, Helsinki, Finland (12/18/2014). Informed consent was obtained from all participants. The present study was undertaken as a follow-up evaluation among those enrolled in a sleep recording.

A total of 20 retained patients constituted the present follow-up study group. Of them, 13 had been included in an overnight polysomnography study previously, as reported by our group in 2018 [7]. Additional 4 patients, who had undergone a comprehensive overnight polysomnography in a sleep laboratory using identical methods [7], were included in this study. The remaining 3 participants had previously undergone ambulatory polysomnography (American Academy of Sleep Medicine Type III sleep monitor), and the data were gathered from their medical records. Total sleep time was unknown, but at the time of the testing had been deemed sufficient by the treating physician. The scoring guidelines and sleep apnea definition of the American Academy of Sleep Medicine were applied [12]. All patients had an apnea-hypopnea index (AHI) or respiratory event index (REI) ≥ 5 events per hour of sleep.

### Positive Airway Pressure therapy (PAP)

PAP therapy was proposed to all patients, as myofunctional therapy and oral appliance therapy are contraindicated in this patient group frequently suffering from co-morbidities of muscle weakness, joint laxity, and craniofacial anomalies [4, 13, 14]. PAP therapy was initiated mainly at home with an automatic PAP device, APAP (ResMed, Sydney, Australia) according to a protocol previously described [15]. PAP data was obtained from ResScan program (ResMed, Sydney, Australia). Acceptance and adherence were evaluated using medical records, self-reports, and data from CPAP device.

### Questionnaires

Epworth Sleepiness Scale (ESS) was employed to evaluate daytime sleepiness. ESS scale ranges between 0 and 24, where a value of 11 or above signifies pathological sleepiness [16]. A validated self-rating depression questionnaire (DEPS) was applied to identify possible depression. DEPS scale ranges between 0 and 30, where a value of 12 or above is considered a cut-off point for clinical depression [17].

Baseline ESS score, before treatment, was available from 13 patients included in our previous paper [7] and from 4 additional patients. Baseline DEPS data were available from 11 previously reported [7] and from 3 additional patients. The self-evaluation ESS and DEPS questionnaires were repeated in 2019 by mail after a minimum of one year of PAP treatment.

### Statistical analysis

Sample size of 20 met the recommended threshold of 12, allowing dropouts, for a sufficiently precise estimates of mean and variance of variables with continuous outcome in pilot studies [18]. Statistical analyses were performed with SPSS version 23 software. Correlation between the variables was examined statistically with Spearman’s rank correlation test. Relationship between the mean self-reported sleepiness and depression symptoms before and after sleep apnea treatment were compared with non-parametric Mann-Whitney U test.

## Results

### Patient characteristics

Table 1 presents the patients’ characteristics. All the 20 patients (11 females) were ethnic Finns. The age range was 27–77 years (mean age 51 years) at baseline prior to treatment. The participants represented OI types I (*n* = 5), III (*n* = 6), and IV (*n* = 6), as classified according to the original Sillence classification [19]. OI type was unknown in 3 patients. Information on the participants’ genotypes was not available. Average height of the patients was 137 cm and height Z-score ranged between − 14 and 0.1 (median − 4.5). Significant correlations were found between severity of OI (i.e. OI type) and the height of the individual (r_s_=-0.744, *p* < 0.001), and between the severity of OI and BMI (r_s_= 0.725, *p* < 0.001); the more severe the OI, the shorter the individual and the greater the BMI, as expected. Medications acting on the central nervous system and therefore potentially inducing sleepiness were used by two patients; including opioids and anti-anxiety medication.

**Table 1:** Osteogenesis imperfecta (OI) patient characteristics, polysomnography findings, and self-evaluation of daytime sleepiness and depression

Patient	Age (years)	OI type	BMI kg/m2	AHI/hour	ESS		DEPS		PAP therapy		
					baseline	PAP therapy	baseline	PAP therapy	0=no treatment 1= PAP therapy 2=refused 3=no data	% of days with devise usage	Daily usage/hours
1	29	I	23.4	11.4	9	NA	5	NA	0		
2	49	I	33.6	29.5	13	13	14	19	1	91	4.3
3	69	I	20.3	21.0	5	NA	3	NA	1		
4	72	I	21.1	23.1	6	NA	NA	NA	2		
5	77	I	24.9	14.2	6	NA	9	NA	1	100	7.0
6	27	III	37.6	21.4	11	3	NA	1	1	50	7.3
7	34	III	62.1	9.0	1	NA	NA	NA	3		
8	40	III	29.5	10.0	5	3	21	6	1	100	8.5
9	46	III	33.4	7.5	4	3	7	4	2		
10	52	III	43.0	45.1	11	9	6	6	1	73	4.4
11	71	III	32.1	24.8	14	13	11	11	1	14	4.0
12	44	IV	23.7	11.3	6	NA	23	NA	3		
13	45	IV	26.0	11.7	NA	12	NA	23	1	100	7.1
14	49	IV	29.0	18.5	4	3	1	3	1	100	10.0
15	52	IV	22.4	5.8	11	12	6	9	1		
16	58	IV	26.4	24.9	1	3	13	7	1	100	7.4
17	63	IV	26.7	10.7	3	7	4	10	1		
18	35	NA	37.2	111.0	NA	2	NA	NA	1	100	9.2
19	46	NA	31.6	17.0	NA	NA	NA	NA	2		
20	65	NA	31.3	85.0	5	5	2	7	1	100	7.0
Average	**51**		**30.4**	**26.1**	**7**	**7**	**9**	**9**		**84**	**6.8**
SD	15		9.6	27.4	4	4	7	6		28	2.0
Range	27-77		20.3-62.1	5.8-111	1-14	2-13	1-21	1-19		50-100	4-10

*Abbreviations*: OI type I: Mild, non-deforming; OI type III: Moderate to severe, progressively deforming; OI type IV: Moderate with wide variety in severity; Height Z-score: relative height as SD from gender-specific population median; *BMI* body mass index, *AHI* apnea and hypopnea index, *ESS* Epworth sleepiness scale (0–24 score scale), *DEPS* Depression Scale (0–30 score scale), *PAP* positive airway pressure, *NA* not available

Self-evaluation of daytime sleepiness and depression was conducted at baseline and with PAP therapy. The pre-treatment data is partly overlapping with Arponen et al. [7]

The average AHI/REI index had been 26 ±27 per hour of sleep, brought about by almost entirely obstructive apnea (obstructive AHI, known in 15 patients, averaged 23/h) (Table 1). Three patients had severe apnea and eight had moderate apnea. The number of arousals related to the total sleep time was on average 33 with a high deviation of ±24, implying a large variation in the sleep fragmentation. None of the patients had severe nocturnal hypoxemia, defined as greater than 30% cumulative time spent with SpO_2_ below 90% (CT90) [20]. In all but one patient, the mean transcutaneous CO_2_ had remained below 6.5 kPa and therefore nocturnal hypoventilation was excluded. The augmented study group was tested for correlations between AHI and other variables. Again, neither the BMI or ESS score correlated with AHI (*p* > 0.05), as in our earlier report [7].

### Sleep apnea treatment

According to the medical records, all of the patients had been offered PAP therapy one to three months after the diagnosis of OSA. One patient died before therapy initiation. Three patients refused the offered therapy and two interrupted it due to discomfort. Their treatment interruption time point was unknown. One patient later restarted the therapy after receiving a better suited CPAP mask.

### Effects of PAP therapy

At the follow-up time point 13 patients, 6 females and 7 males, reported using PAP therapy, as documented in the medical reports, self-reports, and CPAP-device downloaded data, yielding an adherence rate of 87%. Three of them had type I OI, 4 had type III OI, 4 had type IV OI, and 2 patients an unknown OI type. Their average age was 54 years, and average AHI/REI before treatment 32.9 events/hour (range 10–111). Treatment status of one patient was unknown. Follow-up CPAP reading, available for 6 patients, showed an average residual AHI (PAP-AHI) of 0.7 (±0.5) events/hour. Weight measurements of these patients showed that on average their weight had remained the same as at baseline (*p* = 0.79). For 11 patients, data were available regarding the adherence rates to PAP therapy after on average of 41 months course of therapy (median 30 months, range 13–153 months) (Table 1). Information regarding adherence was derived from CPAP-device downloaded data over the whole treatment duration in half of the patients, and from self-reports in the remaining patients whose treatment was organized by other hospital districts. The average usage time of PAP was 7 ±2 hours/day.

### Daytime sleepiness and depression

**Fig. 1:**
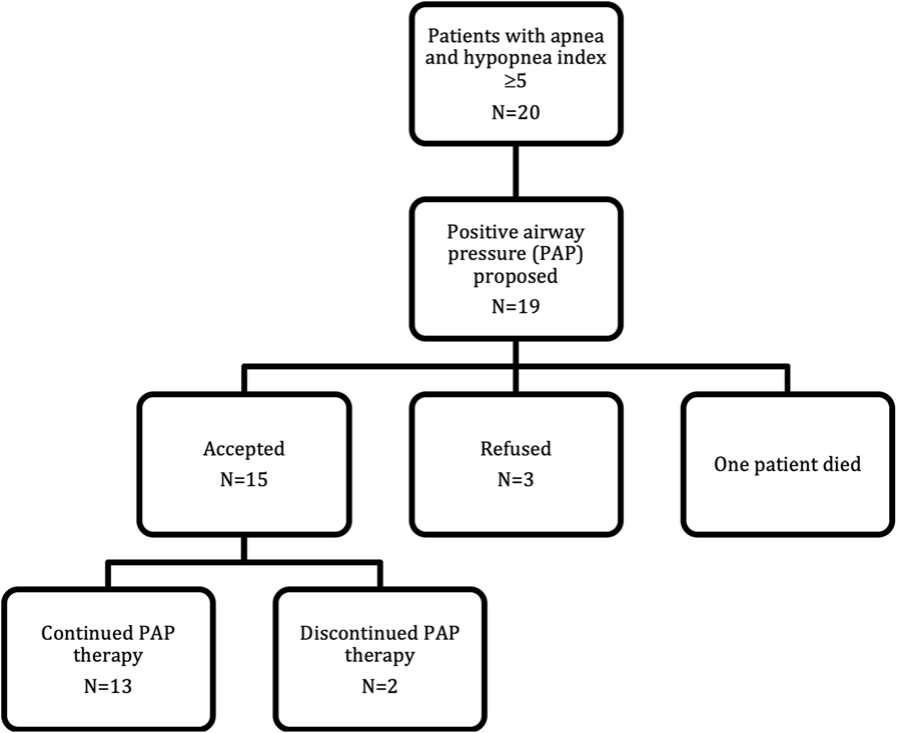
Patient flow diagram

Baseline ESS questionnaire data were available for 85% of the patients. The total number of patients with abnormally high ESS value of 11 or above, indicative of increased daytime sleepiness at baseline, was 5, yielding a 29% prevalence (Fig. 1). The mean score was 7 ±4 in the whole group, men having higher sleepiness scores (8 ±5) than women (6 ±4) but the difference was statistically insignificant (*p* = 0.536).

Baseline DEPS data, available for 70%, indicated in 4 patients (29%) a DEPS score ≥12, a cut-off value for clinical depression (Fig. 1). The mean DEPS score was 9 ±7 in the whole group, and 8 ±4 for men and 10 ±9 for women. The ESS or DEPS scores did not correlate with each other, or with any of the other measured variables (*p* > 0.05).

Follow-up ESS questionnaire was completed by 13 patients (65%), 11 of whom were using PAP (Table 1). At the follow-up, 4 of the 13 respondents reported an ESS score of 12 or 13 indicating excessive daytime sleepiness. Of them, 3 were using PAP as prescribed and one had discontinued treatment. Baseline values were available for 3 of these 4, and they were all above the cut-off value; ranging from 11 to 14, suggesting a persisting excessive daytime sleepiness. Two PAP-users developed from sleepy (ESS > 10) to not-sleepy (ESS ≤ 10), whereas opposite change was not observed. Six patients reported reduced ESS values with treatment and 5 an increased value.

**Fig. 2:**
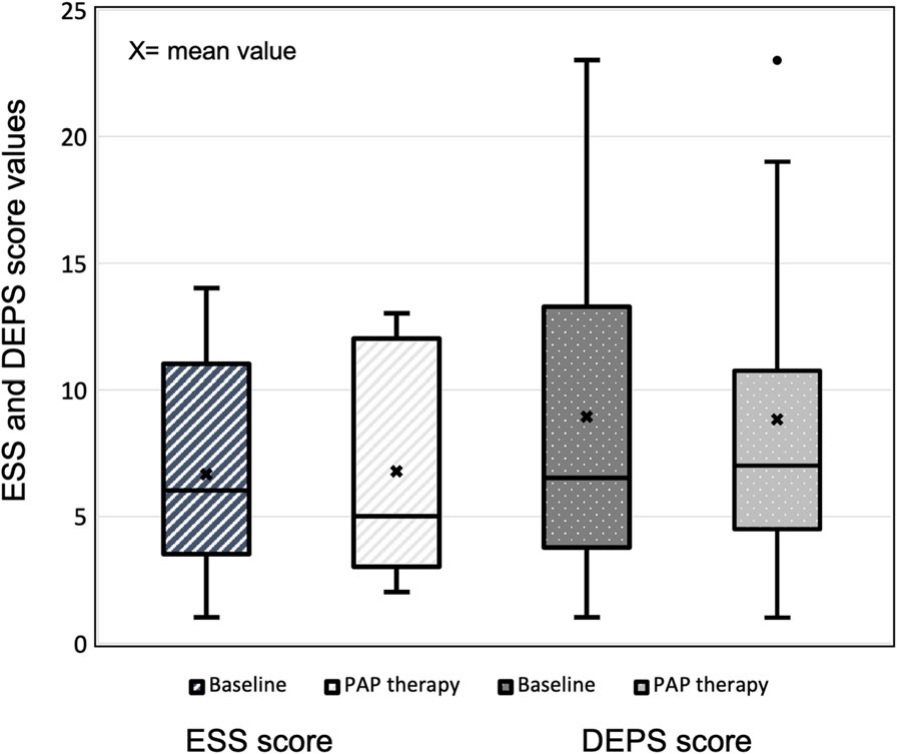
Boxplot of Epworth Sleepiness Scales (ESS) and self-rating depression (DEPS) scores at baseline and after positive airway therapy (PAP) in adult Osteogenesis imperfecta patients with diagnosed sleep apnea

Mean ESS score at follow-up was 7 ±4 (Fig. 2). Among all those 11 patients who completed the ESS questionnaire both at baseline and at follow-up, the largest ESS value increase was 4 and the largest reduction 8, and the median was a reduction by -1. Two patients who had declined from PAP treatment showed minor changes of 1 and − 1 respectively. The mean self-reported sleepiness as expressed by the ESS score remained unaltered with treatment (Mann-Whitney U = 107.5, *p* = 0.902), and the values showed a statistically significant positive correlation between the two evaluation time points on individual level (r_s_=0.63, *p* = 0.038).

Follow-up DEPS questionnaire was completed by 12 patients (60%) (Table 1). In the follow-up questionnaire, 2 patients reported abnormally high DEPS scores of 19 and 23. One of them had received a high DEPS score already at the initial evaluation; baseline data was not available for the other. The first one was a male with type I OI and a mean usage of PAP for 4.3 hours per day for 91% of nights. The other one was a female with type IV OI and daily use of CPAP for 7.1 hours. Both of these patients also reported persistent excessive day-time sleepiness.

Altogether 10 patients replied to both baseline and follow-up DEPS questionnaires. On average, depression symptoms remained unaltered from baseline to follow-up with a mean score of 9 (U = 86, *p* = 0.94) and did not correlate on individual patient level (*p* = 0.395) (Fig. 2). Depression symptom score changes ranged from an increase by 6 to reduction by 15 (median; increase by 1). The two patients, who had declined from PAP treatment, reported a change of 3 and − 3.

At follow-up, ESS and DEPS values correlated positively (r_s_=0.829, *p* = 0.001) implying that the more depression symptoms the patient suffered from, the more tired they felt.

## Discussion

In the present pilot study, we evaluated acceptance of positive airway pressure (PAP) therapy for sleep apnea in a group of OI adult patients, and how self-experienced daytime sleepiness and depression evolved during 1- to 4-year treatment follow-up. To the best of our knowledge this is the first study to evaluate the use of PAP therapy in adult OI population.

### Treatment options for sleep apnea in OI

Treatment of sleep apnea includes lifestyle changes, weight control, a night splint that brings the mandible forward, surgery, or PAP. PAP is the leading therapy and recommended for all patients with moderate to severe OSA (AHI ≥15) [21, 22]. Treatment of mild OSA with PAP was recently reported to improve the quality of life of the patients [23]. In OI patients, a removable appliance for bringing the mandible forward is often contradicted as the patients frequently have a relatively prognathic mandible and may suffer from posterior open bite [13, 24]. Thus a splint therapy would in long term likely accentuate the malocclusion [25]. Surgical advancement of lower jaw or both jaws to increase pharyngeal airway space would likewise be unfeasible in OI [26]. Weight reduction in patients with low ambulatory ability may also be very difficult [27]. PAP treatment therefore is the best and nearly exquisite therapy option for OI patients with sleep apnea.

### Acceptance of PAP treatment

Compliance to PAP therapy is defined as a regular use of the appliance more than 70% of nights for at least 4 hours/night [28, 29]. In our study, 15 out of 18 (83%) patients accepted to initiate PAP therapy. Of them, 13 stayed on PAP therapy over a mean follow-up period of 41 months. This is, by far, better PAP adherence than previously reported in non-OI patients [28]. Nonadherence to PAP, defined as nightly use of less than 4 hours, has been observed in 46 to 83% of sleep apnea patients in the general population [29]. Discontinuation rate of 5–50% has been documented in the general population, most frequently occurring during the first months of use [28]. In our OI patient group, the discontinuation rate was 13%.

### Effects of PAP treatment on day‐time sleepiness and depression

Past studies have demonstrated that a third of patients in the general population are still sleepy after effective OSA treatment, when a success has been defined as an AHI value below 5 [30, 31]. In our study on OI, daytime sleepiness, as expressed by ESS, remained unaffected, on average, with treatment.

A high prevalence of depression among OSA patients in general population has been suggested, although the findings are inconsistent [9, 32]. Analysis of the relationship is complicated by the fact that sleep apnea and depression have overlapping symptoms as well as predisposing factors, such as obesity [33]. Certainly, poor quality of sleep affects mood through neurotransmitter dysfunction and/or possibly also through the impaired immune response caused by hypoxia in OSA. On the flip side, conflicting results of OSA treatment on reducing depression have been reported [9, 32, 34]. In our study among adults with OI, frequency of self-reported depressive symptoms remained unaffected by sleep apnea treatment. Only a few papers have previously studied depression in adults with OI. They report, in certain OI patient groups, greater self-reported level of depression as compared to the general population [35, 36].

A growing interest has been directed towards the concepts of listening effort and fatigue, social isolation, and depression that are associated with hearing loss [37–39]. In individuals with OI, hearing loss is more pronounced in mild types and becomes more severe with advancing age [37–40]. We found more reports of severe fatigue among those adults with mild OI type as compared to those with severe OI type [6], a finding which might be explained partly by fatigue caused by listening effort. Moreover, there is some evidence that OSA further affects hearing functions negatively [37].

### Limitations of the study

We recognize that our study was limited by the small study cohort and variable follow up data. Missing data reduces the power of the analysis. A shortcoming in assessing the effect of the PAP treatment on the subjective sleepiness is that we did not have polysomnographic data on the treatment results of all the patients. Self-reported usage of CPAP has been estimated to include an overestimation of 1.2 hours/day on average [41]. This would theoretically exclude one of our patients from the target appliance adherence. In addition, our current findings on a small patient group with relatively low average level of sleepiness might not be representative of the whole population. Further longitudinal studies, with larger study population including patients with varying severity of OSA and symptoms, would be useful to affirm our findings.

## Conclusions

Osteogenesis imperfecta patients suffering from obstructive sleep apnea accepted PAP therapy well and showed excellent adherence to it. Nevertheless, we found that severity of self-evaluated daytime sleepiness and depression did not reduce, in general, with treatment of OSA. In patients with severe chronic conditions or deformity, the complexity of background factors behind sleepiness and possibly depression may be reflected as unexpectedly poor subjective improvement of symptoms by PAP therapy. Further studies with larger patient cohorts are needed to further elucidate the role of PAP in OI management.

## Data Availability

The datasets supporting the conclusions of this article are included within the article. Polysomnographic records and clinical patient examination records are available from the corresponding author on reasonable request. I can confirm I have included a statement regarding data and material availability in the declaration section of my manuscript.

## Abbreviations

AHI: Apnea-hypopnea index
BMI: Body mass index
CPAP: Continuous positive airway pressure
PAP: Positive airway pressure
DEPS: Self-rating depression questionnaire
ESS: Epworth sleepiness scale
OI: Osteogenesis imperfecta
OSA: Obstructive sleep apnea
REI: Respiratory event index
